# CD97 expression level and its effect on cell adhesion in Preeclampsia

**DOI:** 10.1186/s12884-022-05280-z

**Published:** 2022-12-26

**Authors:** Ayhan Atigan, Semih Tan, Hulya Cetin, Omer Tolga Guler, Saim Ozdamar, Yeliz Arman Karakaya

**Affiliations:** 1 Department of Obstetrics and Gynecology, Faculty of Medicine, Karabuk University, School of Medicine, Karabuk, Turkey; 2 Department of Histology and Embryology, Faculty of Medicine, Pamukkale University, Denizli, Turkey; 3 Department of Obstetrics and Gynecology, Faculty of Medicine, Pamukkale University, Denizli, Turkey; 4 Department of Pathology, Faculty of Medicine, Pamukkale University, Denizli, Turkey

**Keywords:** Preeclampsia, Cadherins, CD97, E-cadherin, N-cadherin, Integrin beta-4

## Abstract

**Objectives:**

Cellular interactions and cell adhesion underlie preeclampsia (PE). The aim of the current study is to investigate the role of cell adhesion molecules such as CD97, neural (N)-cadherin, epithelial (E) -cadherin and integrin beta-4 in PE.

**Methods:**

This prospective study included 20 pregnant women with PE and a control group of 16 healthy pregnant women who were matched for age, gestational age, gravida and parity. Standard blood tests and placental cell adhesion molecule immunohistochemical staining were examined.

**Results:**

The creatinine, uric acid and lactate dehydrogenase (LDH) levels from standard blood tests were found to be statistically higher in the PE group (*p* = 0.002, *p* = 0.000, *p* = 0.001; respectively). In the PE group, the CD97 maternal serum level was statistically significantly lower, as was its immunohistochemical expression in placental sections (*p* = 0.028, *p* = 0.000; respectively). The E-cadherin expression score was statistically higher in the PE group compared to the control group (3,65 ± 1,84 vs 2,06 ± 1,76 respectively; *p* = 0.003). The N-cadherin expression score was statistically lower in the PE group compared to the control group (1,50 ± 0,82 vs 2,43 ± 1,59 respectively; *p* = 0.049). Integrin beta-4 was not statistically different between groups.

**Conclusions:**

Cellular interaction may be responsible for PE as in cancer. A balance in intercellular communication, as researched in cancer therapy, may offer the solution in PE.

## Introduction

Preeclampsia (PE) affects about one in ten pregnancies and is one of the most important causes of maternal and fetal morbidity and mortality. Preeclampsia is a disease specific to pregnancy and its pathogenesis has not been fully elucidated. Clinical symptoms are seen after 20 weeks of gestation as the sudden onset of hypertension (≥ 140/90 mm Hg), proteinuria (≥ 300 mg/24-h; ≥ 0.3 protein/creatinine ratio in spot urine; dipstick reading of 2 +), edema and often fetal growth retardation [1]. In the absence of proteinuria in new-onset hypertension, the presence of any of the following also leads to the diagnosis: thrombocytopenia, renal failure, impaired liver function, pulmonary edema or new-onset headache and visual symptoms [1].

Post-implantation of the blastocyst in the uterus, cytotrophoblast cells proliferate evermore and turn into multinucleated syncytiotrophoblast cells. The syncytiotrophoblasts form maternal blood uncouple from fetal blood, and provide chorionic villi attachment [2]. The changeover between proliferation and infestation phenotypes consists owing to changes in the levels of growth factors and cell adhesion molecules. Adhesion, proliferation and migration transactions are considerable for early pregnancy and the build up of a healthy placenta [3]. Although the etiology of PE has not been fully elucidated, placental defects may be due to insufficient or incomplete trophoblast cell invasion [4]. There is an increase in maternal placental vascular endothelial permeability [5]. The main causes of endothelial dysfunction are decreased or dysregulated expression of endothelial cell attachment proteins [6]. Changes in cell phenotype between epithelium and mesenchyme, epithelial mesenchymal transition (EMT) and mesenchymal epithelial transition (MET) are considerable in organogenesis and complex shaping of the embryo during pregnancy, and in metastasis of most carcinomas [7]. EMT is a process in which epithelial cells lose adhesion, increase motility, and change to a mesenchymal phenotype [8]. There are molecular signals that have a considerable role in the EMT process.

Cadherins are members of calcium-dependent molecules and have prominent key roles in cell differentiation and adherence. Tissue modeling, robustness and homeostasis are ensured by the interaction of these transmembrane proteins. E-cadherin (Epithelial cadherin), an eminent cell–cell adhesion molecule, is expressed in villous cytotrophoblasts. However, they disappear when they differentiate into syncytia. E-cadherin is negatively correlated with trophoblast cell invasion. In this way, it causes the absence of E-cadherin in the syncytiotrophoblast of the ordinary first and second trimester placenta. When the literature is examined, the information about the relationship between PE and E-cadherin is controversial. It is predominantly in the direction of increased expression of E-cadherin. The necessity of further studies has been emphasized in previous studies [9–12].

N cadherin (Neural cadherin) is a mesenchymal classical type I cadherin. Its effect on cell invasion in various cancers is well described. N-cadherin is responsible for the aggressive progression of solid tumors. It is also known as cadherin 2 because of the increase in cadherin 2 gene expression in multiple myeloma patients for whom N-cadherin is held responsible [13]. In recent studies, N cadherin has been shown to play a role in trophoblast invasion and PE [14–16].

Integrins are transmembrane receptors in protein structure. These receptors, which are made up of two subunits (α and β), mediate the connection between cells and the extracellular matrix (ECM). The integrin beta-4 unit pairs with the α6 subunit (α6β4-integrin) to form a receptor for laminin and participates in the structure of hemidesmosomes. It takes part in cell proliferation, migration and invasion processes, respectively [17–19]. Integrin beta-4 overexpression in assorted types of metastatic cancer has been connected with a poor prognosis and low survival [20, 21]. A decrease in the amount of integrin α6β4 complex and an increase in the α1β1 component have been shown during classical trophoblast invasion [22]. Interstitial and endovascular invasion is not sufficient in PE [23].

CD97 is a protein encoded by the ADGRE5 gene. CD97, also known as BL-Ac[F2] is associated with one of the epidermal growth factor seven transmembrane (EGF-TM7) family of class II TM7 receptors [24]. In a study examining its structural and functional characterization, CD97 was thought to be involved in cellular adhesion by interacting with the ECM and other cell surfaces [25]. Three known ligands of CD97 are decay accelerating factor (DAF, CD55), integrin α5β1 and chondroitin sulfate that is an ECM component [26–28]. The information obtained shows that CD97 participates in tumor differentiation, migration, invasion and metastasis [29].

The main goal of the present study is to contribute to the understanding of the effect of CD97, E-cadherin, N-cadherin and integrin beta-4 expressions immunohistochemically in PE patients and normal term placental tissue and to explore the expression level of CD97 in serum.

## Material and methods

Patients who were hospitalized for delivery between July 2019–2020 at Pamukkale University Medical Faculty Hospital Gynecology and Obstetrics Clinic were included in the study. Women who did not have diabetes, vaginal infection or other systemic disease in the PE group, whose blood pressure was higher than 140/90 mm Hg when measured at different times (6-h interval), and protein more than 300 mg in 24-h urine (or equivalent measurements) were included (*n* = 20). ACOG guideline was used when diagnosing preeclampsia [1]. The control group had 16 normotensive women who had no health problems. The placentas in our study were collected within one hour after cesarean or vaginal delivery. The fetal and maternal surfaces of the placenta were cleaned and examined. Two samples were taken from both surfaces, the margin region of the placenta and the middle region between the center and the margin. They were followed by immunohistochemical staining. In order to determine the serum CD97 level of the pregnant women, 5 ml blood samples were taken from each patient after obtaining the consent of the patients. The Pamukkale University Scientific Research Projects Coordination Unit funded our study after ethical approval (project number. 2019HZDP021).

### Preparation and ımmunohistochemical staining of tissue sections

10% Neutral buffered formalin (NBF) fixed parrafin embedded (FFPE) placenta tissues cut at 5 µm thickness by rotary microtome. Parrafin disolved from the tissues by xylen. After deparaffinization, xylene is removed with 100% ethanol and the tissues are rehydrated through a descending series of alcohol to water. With sodium citrate buffer, the heat-induced epitope retrieval method was utilized (10 mM sodium citrate, 0.05% Tween 20, pH 6.0). Incubating the tissue with 3% hydrogen peroxide (H2O2) for 10 min inhibits endogenous peroxidase activity. The tissue slides were incubated overnight at 4 °C with polyclonal antibodies against E-cadherine (Bioasssay Tech. Lab. BT-AP02809, Dilution rate: 1/100, Birmingham, United Kingdom), N-cadherine (Bioasssay Tech. Lab. BT-AP05798, Dilution rate: 1/200, Birmingham, United Kingdom), Integrin 4 (Fine Test, Fine Biotech. Co., Ltd, FNab04351, Dilution rate: 1/100, Wuhan, China) and CD97 (Fine Test, Fine Biotech. Co., Ltd, FNab01510, Dilution rate: 1/100, Wuhan, China). DAB (3,3′-Diaminobenzidine) used for chromogen staining and hematoxylen utilized for counterstaining.

### Elisa method

The indirect elisa method was used to quantitatively detect CD97 serum levels. Each serum sample of the individuals and the standard reagents of the CD97 elisa kit were analysed in duplicate with the CD97 ELISA kit (Fine Test, Fine Biotech Co. Ltd., EH7229, Wuhan, China) as per the guidelines provided by the manufacturers. Each assay's absorbance was measured using a plate reader (Promega, GloMax®-Multi Detection System, USA) at a wavelength of 450 nm.

### Semi-quantitative assessment

All the immunostaining slides were examined in a blinded approach by three independent observers. Integrin beta 4, E-cadherin and N-cadherin showed membranous staining. CD97 immunoreactivity was observed in the cytoplasm. Using the ImageJ technique, staining intensity and the proportion of positively stained cells were determined by capturing 15 picture samples from each sample under the microscope (X40). The percentage of positive cells was graded as 0 (˂10%), 1 (11%–50%), 2 (51%–75%), and 3 (> 76%), whilst the intensity of staining was scored as 0 (negative), 1 (weak), 2 (moderate), and 3 (strong). The intensity x percentage staining score formula was used to transform the immunohistochemical staining expressions of E-cadherin, N-cadherin, Integrin beta-4, and CD97 that were included in the statistics. Semi-quantitative analysis resulted in a final score that varied from 0 to 9 [16, 30–32].

### Statistical analysis

Software SPSS 21.0 (SPSS Inc., Chicago, IL) was used for the statistical analysis. The power analysis was performed by G-Power version 3.1.9.6 application. Quantitative data were expressed as mean value ± Standard Deviation (SD). Semi-quantification of immunohistochemical staining scores was analysed by a Mann Whitney U Test. The comparison between E-cadherin, N-cadherin, integrin beta-4 and CD97 expression and clinicopathological features was analyzed using the Pearson test. Statistical significance was designated as *p* < 0.05.

## Results

When evaluated in terms of gestational week, gravida, parity and age, the control and preeclamptic groups were similar. As anticipated, the preeclampsia group's systolic and diastolic pressures were much higher than those of the control group (Table 1).

**Table 1:** Clinical traits of preeclamptic pregnant women and the control group

	Control (normotensive) (*n* = 16)	Preeclampsia (*n* = 20)	*p* value
Gestational week	32,5 ± 4,4	34,2 ± 3,7	0.214
Age	28,9 ± 7,2	32,8 ± 6,6	0.104
Systolic Pressure	112,5 ± 14,4	152 ± 13,6	0.000 *
Diastolic Pressure	69,0 ± 8,7	92,0 ± 6,1	0,000 *
Gravida	2,5 ± 1,4	2,4 ± 1,3	0.937
Parity	1,1 ± 1,0	0,8 ± 1,0	0.249

^*^ *p* < 0.05; statistically significant

Serum CD97 levels of preeclampsia (0.271) and control groups (0.346) were compared statistically. The preeclampsia group's value was found to be considerably lower (*p* = 0.028). The preeclampsia group had statistically significantly higher levels of creatinine, uric acid and lactate dehydrogenase (LDH) (Table 2).

**Table 2:** Standard blood tests and CD97 serum level of control and preeclamptic groups

	Control (normotensive) (*n* = 16)	Preeclampsia (*n* = 20)	*p* value
CD97 (µmol/L)	0,346 ± 0,10	0,271 ± 0,87	0.028 *
Creatinine (mg/dL)	0,58 ± 0,12	0,74 ± 0,19	0.002 *
AST (IU/L)	16,7 ± 7,5	25,9 ± 16,6	0.077
ALT(IU/L)	13,2 ± 11,3	17,3 ± 13,7	0.211
Calcium (mg/dL)	8,8 ± 0,5	8,6 ± 0,6	0.262
Uric acid (mg/dL)	3,6 ± 0,9	5,4 ± 1,4	0.000 *
LDH (U/L)	209,0 ± 55,8	299,5 ± 127,3	0.001 *
ALP (IU/L)	101,3 ± 43,1	132,3 ± 54,9	0.077
PLT (K/uL	244,8 ± 87,5	223,2 ± 76,1	0.459

*AST* Aspartate aminotransferase, *ALT* Alanine aminotransferase, *LDH* Lactate dehydrogenase, *ALP* Alkaline phosphatase, *PLT* Platelets

^*^ *p* < 0.05; statistically significant

**Fig. 1:**
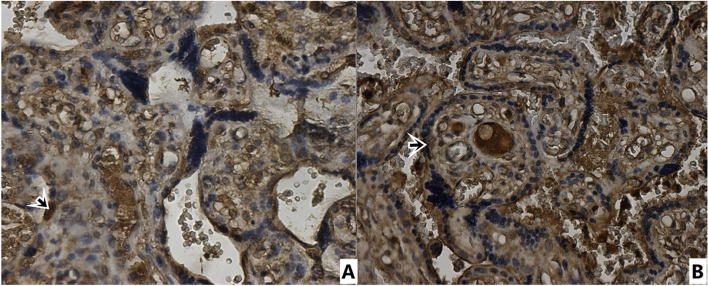
Immunohistochemical staining with CD97 antibody. 40 × magnification. **A** Control Group **B** Preeclampsia group

**Fig. 2:**
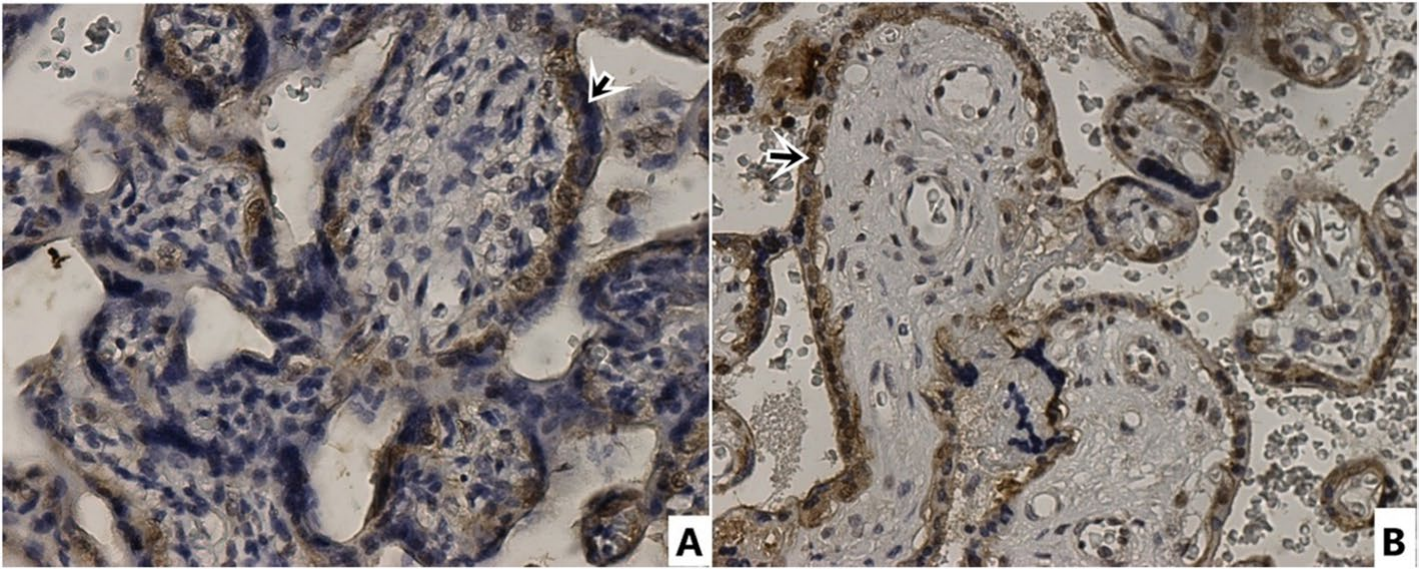
Immunohistochemical staining with E-cadherin antibody. 40 × magnification. **A** Control Group **B** Preeclampsia group

**Fig. 3:**
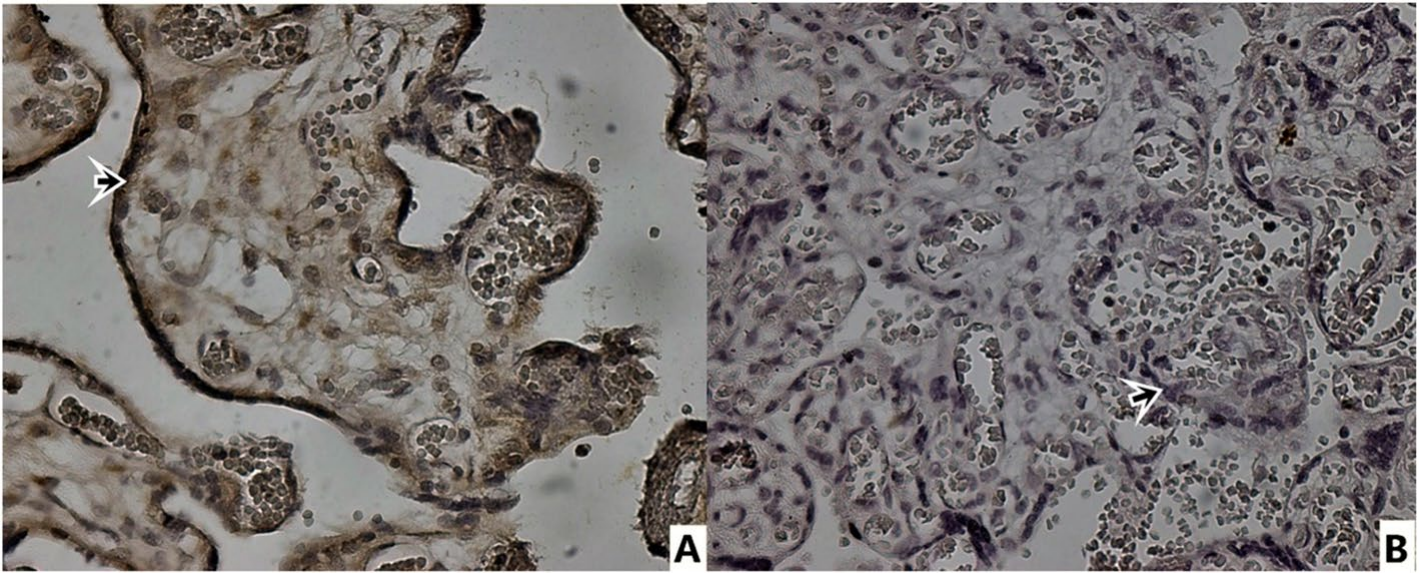
Immunohistochemical staining with N-cadherin antibody. 40 × magnification. **A** Control Group **B** Preeclampsia group

**Fig. 4:**
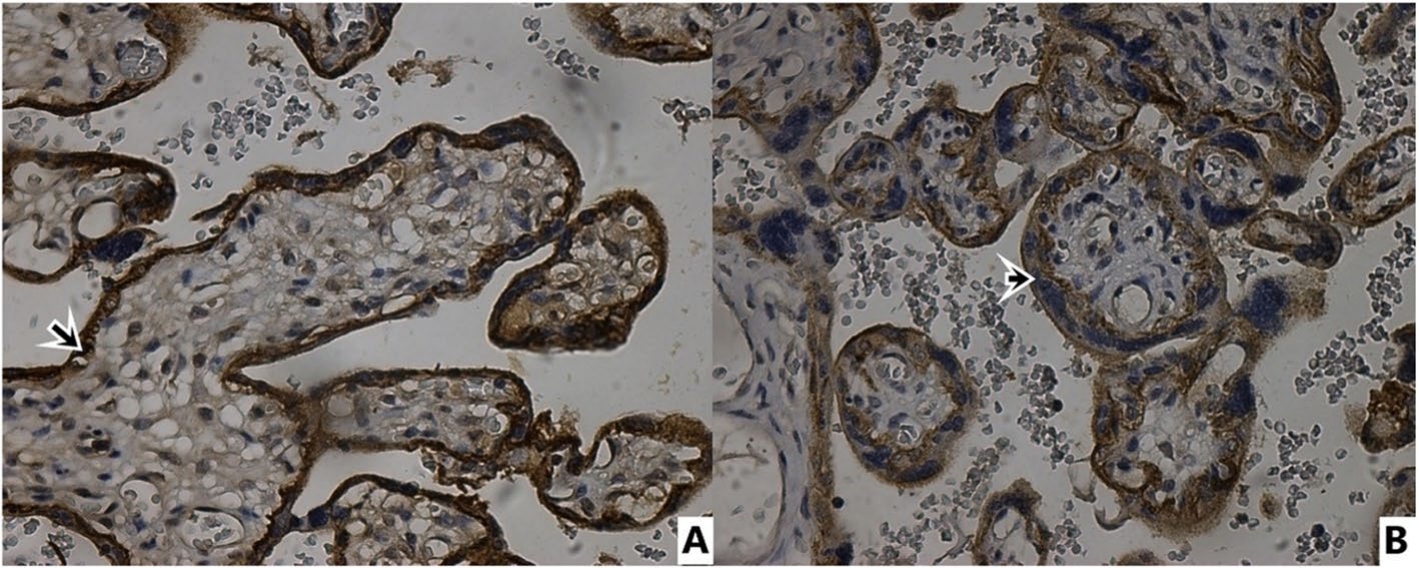
Immunohistochemical staining with integrin beta-4 antibody. 40 × magnification. **A** Control Group **B** Preeclampsia group

In Table 3, CD97, E-cadherin, N-cadherin and Integrin beta4 immunohistochemical staining total scores of placental sections are presented. CD97 expression immunohistochemical (Fig. 1) positive cell staining total score was statistically significantly lower in syncytiotrophoblasts of placentas from preeclamptic pregnant women than in syncytiotrophoblasts of normotensive pregnant placentas (*p* = 0.000) (Table 3). E-cadherin immunohistochemically showed more positive staining in syncytiotrophoblasts of placentas from preeclamptic pregnant women than in syncytiotrophoblasts of normotensive pregnant placentas (Fig. 2). In terms of total score, the preeclampsia group's positive cell staining as determined by immunohistochemical analysis of E-cadherin was statistically substantially higher than that of the normotensive group (*p* = 0.003) (Table 3). In contrast to E-cadherin, N-cadherin showed less positive immunohistochemical staining in syncytiotrophoblasts of placentas from preeclamptic pregnant women than in syncytiotrophoblasts of normotensive pregnant placentas (Fig. 3). Compared to the normotensive group, the total score of immunohistochemically positive cell staining of N-cadherin in placentas belonging to the preeclampsia group was statistically significantly lower (*p* = 0.049). The result of the immunohistochemical staining for Integrin beta-4 (Fig. 4) did not show a statistically significant difference between the groups (Table 3).

**Table 3:** CD97, E-cadherin, N-cadherin, Integrin Beta4 immunohistochemical staining scores of placental sections

Staining	Control (normotensive) (*n* = 16)	Preeclampsia (*n* = 20)	*p* value
CD 97	3,38 ± 2,02	1,10 ± 0,30	0,000 *
E-cadherin	2,06 ± 1,76	3,65 ± 1,84	0,003 *
N-cadherin	2,43 ± 1,59	1,50 ± 0,82	0,049 *
Integrin Beta4	6,37 ± 1,02	6,30 ± 0,92	0,912

^*^ *p* < 0.05; statistically significant

## Discussion

Uric acid and LDH as essential biomarkers, which have prognostic importance for the severity of preeclampsia, were observed in accordance with the literature [33]. As well as the impacts of these fundamental biomarkers, both mesenchymal markers like N-cadherin and epithelial markers like E-cadherin have roles in EMT. CD97 is a protein that belongs to the epidermal growth factor (EGF) family of class II seven transmembran (TM7) receptors that have triplet (EGF1,2,5), quadruple (EGF1,2,3,5), or quintuple (EGF1,2,3,4,5) repeated EGF-like domains [34]. Numerous different cell types express CD97. Although it was initially shown to be expressed by hematopoietic cells, it has also been associated with gastric cancer, thyroid carcinoma, and colorectal cancer in subsequent studies [35, 36]. Overexpression of CD97 contributes to trophoblast cell invasion. In the literature, this issue has been studied very limitedly. Similar to the study of Shen et al. [37], we showed that CD97 immunohistochemical staining of placental sections was less in the PE group. In our study, maternal blood serum levels of CD97 elisa were consistent with CD97 immunohistochemical staining of placental sections. CD97 expression was found to be statistically significantly lower in the PE group. We think that the placenta is not a closed box in patients with preeclampsia and that placental markers can completely affect the pregnant woman. Therefore, in this current study, we showed that in patients in whom we observed less CD97 expression in the syncytiotrophoblast layer, it was excreted less in maternal serum.

E-cadherin placental expression has been found to decrease from the first to third trimesters [38]. E-Cadherin expression was found to be lower in preeclampsia in a different study [11]. As in our study, there are studies showing that E-cadherin expression is increased in preeclampsia and that this causes cytotrophoblast proliferation [9, 10]. Additionally, studies that support the opposition have been published in the literature [11, 12]. In the study of Xu B et al. [12], it was shown that E-cadherin level did not change when TNF-α was induced and trophoblast invasion was reduced. The most significant molecular signs of EMT are N-cadherin gain as well as vimentin, but E-cadherin loss [8]. There are studies showing that E-cadherin expression is increased in preeclampsia and that this causes cytotrophoblast proliferation. E-cadherin, which correlates negatively with trophoblast cell invasion, is elevated in trophoblast cells in patients with preeclampsia [9]. Fedorova et al. found that E-cadherin levels in human preeclamptic placentas were significantly higher than in control placentas [39]. The quantity of E-cadherin in the trophoblasts of preeclamptic placentas is high, and it has been established that this decreases trophoblast cell invasion [9]. In the study of Wang et al., they found an increase in E-cadherin expression and a decrease in N-cadherin expression in the PE group [40]. In the current study, we observed high levels of E-cadherin in preeclamptic placentas. The result of increased e-cadherin levels is consistent with prior research findings. This suggests that the consequent may be associated with limited trophoblast invasion.

N-cadherin, which was shown in neuronal tissues in the first studies in the literature, is also mentioned in most recent mesenchymal studies. Although there are studies on its relationship with EMT, there are rare studies describing the role of N-cadherin in the placenta. Li's study found no difference in N-cadherin expression in both PE and normotensive placentas [16]. N-cadherin expression in PE has been shown to be down-regulated in primary trophoblasts [41]. Lower N-cadherin levels were found in PE placentas compared to controls in the current study. Cancer and preeclampsia are two big dilemmas waiting to be treated in medicine. Tumor cells and trophoblasts have similarities in their proliferation and invasive characterization. Since N-cadherin is held responsible for cancer metastasis, the therapeutic effect of n-cadherin antagonists is being investigated in oncology. In this regard, we feel that N-cadherin antagonists are worth investigating therapeutically for PE, which currently lacks a definite therapy option.

When the villous cytotrophoblast integrates into the syncytium, the aged or damaged multinucleated syncytiotrophoblast passes through the maternal vascular compartment. According to one study, the decreased placental perfusion seen in preeclampsia affects the turnover of villous trophoblast cells [4]. Integrin beta-4 participates in hemidesmosomes responsible for cell proliferation, migration, and invasion [17–19]. Due to a lack of invasion in PE, cytotrophoblasts fail to increase α1β1 and decrease α6β4 [23]. In our study, integrin beta-4 was tend to be significantly expressed in syncytiotrophoblasts. However, there was no significant difference in the immunohistochemical expression of Integrin beta-4 between PE and normotensive placentas. Our study demonstrates that an increase in cytotrophoblast proliferation in PE would not be caused by Integrin beta-4.

The biggest limitation of this study is the small number of patients due to the limited support budget for the research. Therefore, the serum levels of all markers in the placenta could not be examined.

## Conclusion

If a cure for preeclampsia is to be found, the key at this point will be in its histopathology. Cancer, which has been studied by the entire medical field, is comparable to trophoblast invasion. We investigated cadherin, integrin and CD97 involved in cellular adhesion as the essential parts of trophoblast invasion. The serum level and placental expression of CD97, which we think is effective in cell adhesion, were low in the PE group. In the immunohistochemical expressions of PE group placental sections, E-cadherin was increased and N-cadherin was decreased, which was associated with EMT. There was no difference in terms of integrin beta-4. We believe that cell adhesion molecules, which have a place in cancer treatments, may be effective in the treatment of PE with a similar mechanism. As a result, clinical-therapy studies are required as a follow-up to this research.

## Data Availability

The datasets generated and/or analysed during the current study are not publicly available due to privacy/ethical restrictions as they contain personal data but are available from the corresponding author on reasonable request.
